# Meanings and Experiences of End-of-Life Patients and Their Family Caregivers in Hospital-to-Home Transitions: A Constructivist Grounded Theory Study

**DOI:** 10.3390/ijerph192012987

**Published:** 2022-10-11

**Authors:** Eleandro Prado, Sonia Marcon, Luciana Kalinke, Marcelle da Silva, Mayckel Barreto, Angelica Takemoto, Marcela Birolim, Carlos Laranjeira

**Affiliations:** 1 Postgraduate Program in Nursing, Nursing Department, State University of Maringá, Maringá 87020-900, Brazil; 2 Nursing Department, Federal University of Paraná, Curitiba 80210-170, Brazil; 3 Anna Nery Nursing School, Federal University of Rio de Janeiro, Rio de Janeiro 21941-901, Brazil; 4 Nursing Department, Guairacá University Center, Guarapuava 85010-000, Brazil; 5 School of Health Sciences of Polytechnic of Leiria, Campus 2, Morro do Lena, Alto do Vieiro, Apartado 4137, 2411-901 Leiria, Portugal; 6 Centre for Innovative Care and Health Technology, Rua de Santo André 66-68, Campus 5, Polytechnic of Leiria, 2410-541 Leiria, Portugal; 7 Research in Education and Community Intervention, Piaget Institute, 3515-776 Viseu, Portugal

**Keywords:** end-of-life, patients, family caregivers, grounded theory, palliative care, home care, transitions

## Abstract

This study explored the meanings and experiences of patients with terminal chronic diseases and their caregivers, who face the imminence of death in the home environment after hospital discharge. The qualitative study used constructivist grounded theory. The participants were individuals with a terminal chronic illness, discharged to home, and their family caregivers. Data were gathered from in-depth interviews and field notes, and a comparative analysis was conducted to identify categories and codes, according to Charmaz’s theory. The sample consisted of 21 participants. Three inter-related data categories emerged: “Floating between acceptance and resistance: Perceiving the proximity of death”, “Analysing the end from other perspectives: it is in the encounter with death that life is understood” and “Accepting the path: between the love of letting go and the love of wanting to stay”. The categories translate the reconstruction of those facing end-of-life occurring in the home environment. It is amid the imminence of death that life gains intensity and talking about the finitude of life configures an opportunity to see life from other perspectives. Giving voice to individuals facing the mishaps of a terminal illness fosters the path to a comfortable death. For health professionals, it is an opportunity to provide structured and humanized care with an ethical attitude, in defence of human dignity.

## 1. Introduction

Life expectancy is rising globally [1]. By 2060, “an estimated 48 million people (47% of all deaths globally) will die with serious health-related suffering”, which will increase “most rapidly among people aged 70 years or older” [2] (p. e883). A high proportion of these people may require palliative care, “which aims to improve the quality of life of patients facing terminal illness by relieving pain and other distressing symptoms” [3] (p. 18). Palliative care is critical to integrated and people-centred health services [4], with emphasis on home care. Seow and Bainbridge [5] pointed out some domains for quality palliative home care, such as symptom management, integrated teamwork, timely responsive care, and patient and family preparedness.

Since the perception of approaching death can pose profound existential challenges, preparing for death is one of the most important concepts regarding End-of-Life (EoL) care. From an anthropological point of view, death is characterized by the EoL, while the dying process is defined as the interval between birth and death, which means that human life is constantly threatened by death [6]. The awareness of the nearing end of one’s life, and hence a loss of coherence and orientation, necessitates a process of psychological self-adjustment [7].

Resisting change and avoiding one’s mortality can lead to dysfunctional coping mechanisms and increased suffering [8], whereas an accepting attitude can reduce worries and help guarantee a good death [9]. According to current research, discussing death-related themes can potentially reduce grief and even create opportunities for personal growth [10]. Individuals can develop a tolerant attitude toward death through a process of adjustment [11] that fosters anticipatory grief, i.e., the experience of grief prior to the loss. A recent review posits that anticipatory grieving safeguards against any sudden experience of death, affecting both the dying experience and bereavement outcomes [12].

Thus, the repercussions associated with the death and dying process justify a holistic and integrated approach that ensures human dignity and quality of life. Published data focusing on EoL care underlines the importance of assessing palliative needs in terminally ill patients and reinforces the importance of addressing the issue of terminality with all those who face this reality [13,14].

Recent research has shown that providing palliative care throughout the course of a serious illness offers a plethora of benefits for patients and families [15]. Palliative care is “increasingly recognised by international health organisations as a cornerstone of global health and there have been strong, coordinated calls to integrate palliative care within health systems” [2] (p. e890). In Brazil, palliative care is mostly provided at hospitals, as it is a component of Primary Health Care (PHC), but only 10% of hospitals have a specialist team for palliative care [16]. Soares et al. [17] argue that the inclusion of palliative care in PHC might address the shortage of specialists and constitute a concrete first step in changing the status quo. While palliative care was initially associated with cancer care, there is currently a significant drive for its inclusion with other chronic illnesses. Although palliative care should be provided in a home setting, Brazilian data show that about 80% of deaths occur in the hospital [16]. A parallel situation was described in several other countries [18,19,20]. The quality of care for those dying is poorer in hospitals than elsewhere [21]. Therefore, invasive procedures should be promptly stopped when death is anticipated, allowing healthcare professionals to switch from a curative to a palliative strategy [22]. Understanding this critical period may also lead to a general improvement in EoL care.

The interface between acute and community care is common, since patients receiving palliative care often express a wish to receive treatment and/or die in the community [15]. Unfortunately, when patients reach the end of their lives, transitions can be hampered by problems of coordination, unmet care needs, insufficient communication, and lack of liaison between acute and community care settings [23,24,25]. Notwithstanding, to reduce the societal and economic costs of the growing number of chronically ill patients “effective collaboration has become a priority for healthcare systems to help in the transition from primarily acute, hospital-oriented palliative care to community-based palliative care” [25] (p. 3383). In this sense, health systems around the world have invested in the quality and improvement of care, combined with the optimization of resources. Transition of care is a prominent element in this strategy. To maximise comfort and minimise the possibility of readmissions, the transition process includes pharmacological adjustment, remote monitoring, availability of specialized care at home, and a concern to identify and meet the needs of patients and their families [26].

Transitioning from hospital to home is a vulnerable stage in the care trajectory of EoL patients [15], yet few studies consider the experiences of both patient and family caregiver in palliative care transitions. People with different palliative care conditions, especially those close to death, may have different interpretations of transition of care, which can be “defined as significant life events that entail the integration of a new situation or circumstance into a person’s life” [27] (p. 125).

A qualitative study interviewed older persons with long-term illnesses in their final year of life and revealed that most of the treatment they received as they transitioned between care settings was typified by inflexibility and a failure of healthcare staff to listen [28]. Other evidence highlighted that the transitional care process is frequently related to sensations of loss and anxiety in patients and families [29]. A paucity of discussion about care goals and a disrespect for personal preferences were also identified [30].

To the best of our knowledge, no research has looked at this transition process in the Brazilian context, which could offer insights into the role of healthcare practitioners in advocating this care transition process. To address this gap, this study asked, ‘how do patients and family carers understand and live the terminally ill process during hospital-to-home transitions?’ The findings may contribute to the delivery of good quality palliative care in hospital-to-home transitions and emphasize the relevance of person-centred care.

## 2. Materials and Methods

### 2.1. Study Design

A qualitative study was conducted utilising the constructivist grounded theory (CGT) framework [31] to evaluate the meanings and experiences that pervade the lives of chronic illness patients and family caregivers who face impending death at home, following hospital discharge. CGT is an excellent tool for explaining social processes and human behaviour [32], and is widely used in nursing, education, and sociology [31]. It uses flexible yet rigorous analytic techniques, promoting researcher reflexivity, whose epistemological viewpoints include constructivism with symbolic interactionist underpinnings [31]. This study was performed and reported following the Consolidated Criteria for Reporting Qualitative Research (COREQ) checklist [33].

### 2.2. Setting and Sample

The study was conducted in southern Brazil (State of Paraná) and involved patients with advanced chronic illnesses and family caregivers, during hospital-to-home transitions. Participants were recruited using a purposive sampling technique. The selection of individuals took place in two different settings, initially in the “Programa Melhor em Casa –PMC” and later through four Basic Health Units (BHU), both regulated by the Brazilian Unified Health System. These places were chosen because both offer home care after hospital discharge to patients with palliative care needs; however, only the former has a multidisciplinary specialized palliative care team exclusively for this purpose.

We included participants if they (a) were adult (at least 18 years old) patients or family caregivers; (b) had the cognitive ability to participate in the study; and (c) were able to speak Portuguese. Patients also had to: (c) be diagnosed with a terminal medical illness, (d) have knowledge of their limited prognosis, and (e) be experiencing a hospital-to-home transition. Individuals were included regardless of age, gender, and primary diagnosis. Patients with an acute disease and undergoing curative treatment or in physical rehabilitation for non-permanent conditions were excluded. Caregivers were excluded if they had less than three months of experience.

As a result, 11 patients and 10 family caregivers participated in the study. This number was determined by theoretical saturation, as recommended by Charmaz [31], that is, the interviewees guided the conceptual exploration until no new evidence was suggested in the analysis. Theoretical saturation contributes significantly to understanding categories and formulating theory, thus completing the inclusion of new participants and the formation of new sample groups [31].

The 21 individuals were organized into two sample groups. The first group included five patients and six family caregivers, totalling 11 participants, who received specialized home care in palliative care (provided by the PMC). The second group included six patients and four family caregivers, totalling 10 participants, who were only assisted by the BHU. Patients and family members of a given group did not belong to the same family unit.

### 2.3. Data Collection

Data collection took place at the homes of patients and family caregivers, from April to November 2021. The initial contact with the sample groups was through the PMC and BHU. The objectives of the study were explained to eligible participants during a home visit scheduled by telephone and, if the invitation to participate was accepted, a second visit was scheduled to proceed with the interview.

The in-depth interview was guided by a semi-structured script with an initial open question: “Tell me about your experience of terminal chronic disease at home?”. Afterwards, other semi-open questions were used to explore the research topic; for instance, “How do you experience the transition from hospital to home?”, “How did you get news about worsening illness?”; “How did you face the main challenges and difficulties?” and “Do you believe you need to make any future plans?”. We modified the interview guide during data collection due to the emergence of new topics. Although the guide was not pilot tested, questions were rephrased throughout the interviews to increase clarity and understanding. Field notes were taken during and after the interviews.

To ensure consistency and accuracy, the interviews were conducted by a researcher with expertise in qualitative-research interviews. The interviewer was a male registered nurse and a PhD candidate at the time of this study. All interviews were conducted face to face in a private home room to ensure privacy and foster confidentiality. There were no repeat interviews. Interviews lasted between 25 min and 1 h 15 min, with an average duration of 40 min, and were audio-recorded and transcribed verbatim.

To ensure that quotations were accurately translated, they were first translated into English and then back into Portuguese. Example extracts were numbered according to the role of the participant (Patient [PT] or Family Caregiver [FC]) and the type of assistance they received (specialized support in palliative care at home [PC] or general support provided by the Basic Health Unit [BHU]) in brackets.

### 2.4. Data Analysis

We undertook a stepwise inductive analysis of the data, following Charmaz’s method of grounded theory analysis [31]. First, data were coded inductively, line by line, using a content analysis method. To guarantee we could make sense of the data, concepts and important phrases were highlighted and put into subcategories. Second, the axial-coding stage allowed us to see the connections between the categories, such as causative, contextual, and intervening circumstances, as well as action/interaction strategies and outcomes. Finally, the core category and its relationships with other categories became apparent (i.e., selective coding). The constant comparison between the coded concepts generated the core category, which is a highly inclusive abstract term. We engaged in an iterative process to develop the codebook, over several meetings with co-investigators. The categories (and the theory itself) were based on the core category, which comprises major phenomena. Indeed, the emerging categories (including the core category) allowed us to construct a comprehensive theory. Theory generation was accomplished through discussions with the project team, the production of summary memos, and the use of diagramming technique [31]. During the analysis process, a schematic representation outlined analytical ideas and was utilized to describe study findings.

### 2.5. Research Rigour and Reflexivity

The research rigour and validity of CGTs are ensured by consistently using the four criteria proposed by Charmaz [31]: credibility, originality, resonance, and usefulness. Interviews were carefully transcribed, detailed field notes were taken, and frequent comparisons and checks were performed to assure study credibility. The originality of the findings was determined by a comprehensive reflexivity process that included writing memos and evaluating the existing literature. To maintain consistency, each participant acknowledged our interpretation of each interview and agreed with emerging categories. The discussion reflects resonance as it strived to make sense of the findings within the framework of prior research and suggest transferability to similar persons and circumstances. Finally, the usefulness criteria were respected by the relevance of the theoretical model’s practical implications in improving palliative nursing care towards a model of person-centredness care in the home environment.

Recently, there has been an increased interest in the literature on researcher reflexivity, particularly in the setting of grounded theory, which is significant in constructivist frameworks since it is a co-constructive process [31]. Our project’s reflexivity enhanced the transparency and credibility of the research process [34]. We used reflexivity to consider and address the existence of researcher interactions at several phases of the research process, including during subject selection or question formulation, the design of the study, data collection, and data analysis, as well as during the writing process.

The research team included nurses working in palliative care (E.P.; S.M.; L.K.) and experts and researchers in qualitative health research with a constructivist epistemological standpoint and with a nursing and palliative care background (M.d.S.; M.B.; A.T.; M.B.; C.L.).

### 2.6. Ethical Considerations

The study was evaluated and carried out in compliance with the Declaration of Helsinki’s principles, and the protocol was authorized by the Research Ethics Committee of the State University of Maringá—UEM (approval n° 4.518.26). Informed consent, including approval for audio recording, was obtained in writing before each interview. Participants were told they could drop out of the research at any moment. They received no remuneration for their volunteer cooperation. Considering the participants’ physical and psychological vulnerability, nursing support was available if participants wished.

## 3. Results

### 3.1. Sample Description

The sample included eleven terminally ill patients, whose ages ranged from 31 to 84 years. The most prevalent clinical condition was neurodegenerative and cancer disease. How long they lived with the prognosis of incurability ranged from 1 to 4 years. There were ten family caregivers (seven daughters; one brother; one wife and one daughter-in-law), with an average age of 52 years, most of them female (*n* = 9), and with a duration in this role ranging from 5 months to 4 years. Table 1 presents a detailed overview of the descriptive characteristics of the study participants.

### 3.2. Findings from Interviews

**Figure 1:**
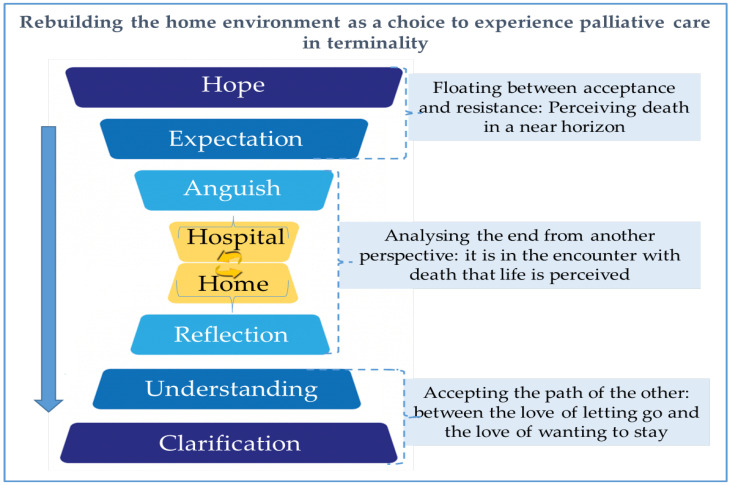
Representative diagram of the terminally ill process during hospital-to-home transitions.

Data analysis identified the categories: “floating between acceptance and resistance: perceiving death in a near horizon”; “analysing the end from another perspective: it is in the encounter with death that life is perceived”; and “accepting the path of the other: between the love of letting go and the love of wanting to stay”, these concepts interact with each other and converge upon this study’s core category: “Rebuilding the home environment as a choice to experience palliative care in terminality” (see Figure 1).

The core category comes from the meanings and feelings experienced by the individual from the moment when terminality becomes an ever-closer horizon. This process begins in the hospital (as an environment that instils unrealistic hopes and expectations in individuals), evolves and culminates in a reflective outcome around the EoL, when care is centred at home.

Amid the fears and anxieties when receiving an unfavourable prognosis, this reflexive process leads to a natural acceptance of the finitude of life, and represents the path that patients and family members go through in the face of imminent death. In this way, understanding death is accentuated as its proximity is admitted. In this context of acceptance, the home scenario proved to be the safest and most satisfying environment.

Patients and family members experience a set of emotional reactions such as hope, expectation and anguish, which are analysed through mental processes that include reflection, understanding and clarification, in a sequenced flow that accompanies the categories under study.

The category “**floating between acceptance and resistance: perceiving death in a near horizon**” reflects the reactions when individuals receive the prognosis of incurability of their clinical condition. It is in this period that feelings of hope and expectation are given new meaning, through behaviours that reveal the desire to be and have a healthy life and the normality that was once experienced.

In general, hope is represented by an inner strength, which apparently weakens in time, because it is based on the prospect of healing. However, with the approach of death, hope becomes based on faith and lifestyle changes, in the expectation of better results. The different focus of hope does not necessarily mean a decrease in hope, as it is a phase of incurable and progressive illness. Through speeches, it is possible to resize hope, that is, to adjust objectives to reality, without this implying the extinction of hope.

*(PT_BHU_1) I was sad to know, because there are so many things that we would still like to do and cannot. But I have faith, the only one who knows things is God, and only He knows about tomorrow.*

*(PT_BHU_4) I don’t know for sure, I just know that it’s difficult, whatever God wants, I can’t really change much, so I left it up to God and them (health professionals), and then I think it will work.*

*(PT_BHU_2) the doctor said that this will not change and that it will not improve, but I think there is no way for him to guarantee that, it may be that with time it will improve.*

When death became an imminent possibility, both sick people and family members persistently hoped for a cure, anchored in their faith in people or previous treatment. However, with the proximity of death, the feeling of hope gradually faded and gave way to frustration and disappointment.

At the same time, returning home was also seen as a setback for hope. The was a perception of failure and frustrated expectation in a healing process that did not occur, and individuals began to more clearly express their fears, apprehension, and uncertainty in the face of what is to come.

*(FC_BHU_3) I already knew deep down that she was in a very serious situation, but even so, our plan was to return home cured […] but hearing that she would not get well, it took me off the ground, it was very difficult and still is.*

*(PT_BHU_5) when they told me that cancer had spread, it felt like I was regressing in life, I was doing so well, but at that moment it seems like it was all in vain, it shook me a lot.*

*(PT_PC_2) My life was getting back to normal, until the moment things got worse, and then everything changed, including her course, but what can you do, that’s what happens to those who are alive.*

The reluctance to naturally understand the severity of the disease and the proximity of death hinders and postpones the acceptance process, generating anguish and emotional overload in individuals. In this context, participants alternated between thinking about the disease and ignoring it and the idea that death might be close. However, the participants assumed the inevitability of death as certain, for themselves and for others.

*(PT_BHU_2) Knowing that [disease] was terrible, we don’t want to, we’re afraid, I didn’t want to go through what my sister-in-law went through, she suffered and had metastases too. They told me that my [cancer] is different, but it is happening too fast too, I didn’t expect it.*

*(FC_PC_3) She is elderly, so we already imagined that, but I don’t know if I’m ready for it [death] now, it’s been a different and very difficult experience, it’s the law of life [sighs] she’s suffering, not only physically, but mentally, she never wanted to be in this situation, maybe for her, it’s a relief.*

Together with the frustration of having one’s plans interrupted, there was also some ambiguity in understanding how best to deal with the terminality of the disease. In this sense, subjects who received follow-up from specialized support networks were less resistant to talking about their condition, which substantially contributed to the understanding of the finitude of life.

In addition, the home environment alleviated feelings of anguish, and participants favoured family life, where they could find protection and support.

The category “**Analysing the end from another perspective: it is in the encounter with death that life is perceived**” translates a new vision established when individuals are ready to understand and accept their finitude, and thus to experience this phase with greater comprehension.

*(PT_PC_3) when I found out about my situation I cried a lot, I didn’t want to understand, I had only one thought. Now I have a new vision of things, I don’t have to keep thinking ahead, I say I’m just thinking about the now, the current moment.*

*(FC_PC_3) today we are much more realistic with the situation, it is heading towards the end, we are also going, we are all going to [die]… there are some people who are closer by nature, this is her case, for this is clear to me.*

Because we are innately spiritual beings, it is natural that every human being seeks meaning, understanding and purpose in life, and that this search is more accentuated when the person is suffering, ill or facing death. Nevertheless, talking about death generates discomfort and fear, and many individuals, although aware of this possibility, prefer not to openly address it, much less mention it, referring to as the “right time”, “the moment “, and “the call”. The use of euphemisms (stylistic resource) is frequent in speeches, as alternative ways of talking about death without ever having to use the word.

*(FC_PC_2) I know it’s going to happen, but we don’t talk about it [death] we don’t like to talk, I realize that she gets a little weird when someone brings it up, and I also think it’s better not to talk, I know she’ll have it in her head all day, and it’s not good for her to dwell on it.*

*(FC_BHU_3) I even think about it [death] sometimes, but my experiences are not good, that’s why I don’t like it, it seems that everything comes to my mind again, I don’t want to spend it with her, so it’s not even good to think about it.*

*(PT_PC_5) it’s our time, but I prefer not to think about it [death] it seems that the more we think, the worse we get, it’s bad to keep it in our head all the time.*

The difficulty in talking about death is expressly related to the clinical history, as this topic was clearly never explicitly addressed by health professionals. Often, the theme of death only emerges when the curative approach is replaced by palliative care, given that from a social and cultural point of view, palliative care is still synonymous with defeat, failure, or even a simple endpoint where nothing else can be done.

In contrast, some participants recognize the EoL and the process of dying as an opportunity for the person to discover new meanings for life, and not just a dark death sentence, empty of meaning and value. In this sense, the final phase of life can contain moments of reconciliation and personal growth.

*(PT_PC_3) Now I have a new view of things, before I wanted to be in public service, and have a good job so I could have a child. Nowadays I know I don’t need all that, I just know that we need to live one day at a time and think about this moment as if it were the last.*

*(PT_BHU_5) I usually say that cancer taught me many things, and one of them was this, the opportunity to stop a little, see things differently, today I pay attention to small details, before I lived a busy life, it was just working, today I know I may not have more time, so I want to enjoy it without worrying about looking at the clock.*

Talking openly about death leads to a comprehensive acceptance of one’s own finitude and provides a reflective opening to life, allowing patients and family members to experience this process more naturally, and consequently with well-adjusted responses, making this stage as painless as possible.

This new look at terminality makes many previously considered essential habits seem superfluous. The day-to-day rush, the accumulation of material goods and the time allocated to others are no longer the priority, that is, the threat of death raises new ways of being, such as enjoying every moment, being beside those you love, dedicating attention to oneself, and giving value and meaning to small details.

When the threat of death is unavoidable, patients and family members are conflicted regarding the acceptance of this process. This is evident in the category: “**Accepting the path: between the love of letting go and the love of wanting to stay**”. This category expresses the ambivalent reactions between relief at the cessation of suffering, and sadness at the departure, and both reactions end up giving meaning to the EoL trajectory.

*(PT_PC_1) we talk about it [death] and I understand it well, I’m not afraid of dying anymore, however, what I think about most now is about her [daughter], because it’s just the two of us, I worry about leaving her alone.*

*(FC_PC_1) I was afraid of that moment [of death], but seeing her in bed, in this condition, it seems that she is no longer there, for me and it is sad, but it must be more painful for her, so I believe, that when she goes [to die], it’s going to be a rest, if she could talk, I’m sure she would want that too.*

Preparing for death entails concluding a life’s work, completing tasks with family and friends, and accepting the inevitable. Death was accepted especially in participants who received specialized follow-up in palliative care. In contrast, individuals lacking this type of support revealed a different acceptance process, without a concrete space to talk about their fears and anxieties. In this sense, accompanying this phase highlights the importance of human care and the importance of compassionate communication by health professionals.

Participants talked about death naturally, including resulting concerns, especially the suffering it can cause. Thus, individuals considered death a cessation of suffering, where suffering is understood as more painful than death itself.

*(PT_BHU_3) one day he will [die]… do what I don’t want is to suffer in a hospital bed, I don’t want that.*

*(PT_PC_5) I’ve been thinking about these things [death] and I only know one thing I don’t want, is to suffer in a bed giving them trouble [family].*

*(PT_BHU_2) My sister-in-law suffered a lot before she died, I don’t want to be like her, I already told her [daughter] that I want to go [die] before suffering.*

The acceptance of death (one’s own or those near to us) is intimately related with the end of suffering (our own or that overloading the family system). When the sick person does not want to leave, the family members who do not want to let their loved ones go. However, when the terminally ill realize death is approaching and the suffering it can generate, the initial resistance gives way to the gradual acceptance and clarification of the inevitable.

*(PT_PC_3) dying is a slow process, I gradually understood, today I can say that I am better prepared, I know there is nowhere to run, so it’s like I say: let’s live better, enjoy, and help others, while there’s still time.*

*(PT_BHU_5) […] what bothers me is not death, it is knowing that I will no longer be here to do what I like, what I have now come to see, I joke that everyone had to experience cancer, just to have this feeling that tomorrow might not be here anymore.*

*(FC_PC_5) obviously, no son wants to say goodbye to his mother, for me he left her there in bed forever, but then I think, did she want that? I’m sure not. Still, I don’t know how I’ll react, if I knew when, I think I wouldn’t want to be there, even though I know it’s for the best.*

Wanting to leave or letting go is a constant discourse in terminality scenarios, however, acceptance is favoured in environments where comfort is a constant. Being ready to leave or letting go results from maturity in understanding the process of dying, it is a courageous way to express gratitude and love in the face of the roller coaster of negative emotions associated with leaving.

## 4. Discussion

This study explored the meanings and experiences that permeate the lives of patients with terminal chronic diseases and their caregivers who experience the imminence of death in the home environment after hospital discharge. Although death is a natural part of human life, our findings indicate a difficulty in facing it naturally. Understanding and accepting death arouses a process of intra and interpersonal reorganization in order to integrate it into their life course. Frequently, “feelings of hopelessness, loss of control and uncertainty around death can have a detrimental impact, particularly in patients with a poor prognosis” [35] (p. 3). Terminally ill patients are aware they are steadily becoming weaker and unable to perform once normal activities [36]. The pain of loss or knowing that the loved one can leave at any time, generates reflections on life and the way of living [37].

In the transition of care between hospital and home, terminally ill individuals experience the process of death and dying in different ways, especially in terms of home environments with specialized care and those where such care is restricted or inexistent. The uniqueness of emotions and reactions to the finitude of life reflects an individual’s social environment and how they interact with it. At this juncture, clearly how death is processed is closely related to the experiences in the family environment and social daily life [38,39].

Although there are different reactions when facing the process of dying, the certainty of a terminal prognosis and an ever-approaching death forces an individual through common phases, changing life priorities and how they view the world. Notably among their choices is wanting to die at home, the preferred place to experience that stage of their lives.

This reality corroborates other studies that also portray the home environment as the scenario of choice to experience the last days of life [40,41]. The home environment, as a second skin, mitigates fears, strengthens interactions and facilitates the proximity of the patient and their family members. Being at home, or in the environment where you want to be cared for, ensures and encourages the sick person’s autonomy, a basic characteristic and one of the most significant benefits of EoL care, as it enables individuals to make decisions and thus maintain take control of their lives [42].

Although the home is recognized in the literature as a place of good death [9,43], both patients and family members can face different stressors, since death still refers to suffering and loneliness [44]. Even though the home is seen as a valued site for both care and death, family caregivers expressed difficulties. In line with other research findings [45,46], many participants did not know what to expect from the dying process, were affected by seeing their family member’s physical decline, and were unprepared for the last hours and minutes before death.

Experiencing this reality and its consequences imply suffering in all human dimensions—physical, psychological, social and spiritual—by the patient and family [47,48]. In these circumstances, health professionals play an essential role in promoting discussion and providing new opportunities for individuals to lead their lives around these limitations [49].

In this study, like others, caring was gendered [50], with most carers being female daughters. Whether care/death at home is appropriate or “ideal”, as indicated by caregivers, must be addressed. A shift to a home death, it might be claimed, can place the strain of optimal palliative care upon family members, frequently female members, and transform the safe haven of home into something else, implying a loss of privacy, worry over the ability to manage, physical fatigue, and, in some circumstances, injury [51].

Kubler-Ross emphasized the value of listening to and supporting the experiences and needs of dying patients, sparking new perspectives on how practitioners might assist terminally ill patients and their families in coping with the knowledge of imminent death [52]. She outlined five stages of coping with dying, namely denial, anger, bargaining, depression, and acceptance. These stages are helpful in describing the emotional process when patients face life-changing illnesses. Individuals can experience the different phases, at various times, without necessarily following a linear order, or not experiencing any of the phases [48]. Identifying and understanding the characteristics of each of these five phases helps health professionals, family members, and above all the sick individual himself to understand the process [53].

In this study individuals tended to experience a set of reactions in a sequential and consecutive order, initiated by hope and expectations, followed by anguish and finally expressed by reflection, understanding and clarification.

During hospital-to-home transitions, hope and expectations emerge as coping strategies that ease the reactions of fear, despair and anxiety, keeping the focus on life [54]. Previous studies reinforced the important contribution that hope represents in the final stage of life [55,56], but also refer that patients and family members commonly express unrealistic hopes or false expectations of cure in this phase [57]. Thus, hopes and expectations must be accepted with some caution and consideration, without forgetting their therapeutic power. Patients who have no expectations of cure or long-term survival can always hope for good symptom control, spiritual peace and a “good death” [58]. For this, there needs to be adequate communication and sharing of the decision-making process, allowing the sick person to make choices, which will facilitate awareness, openness and acceptance of the situation. Confidence in the competence of health professionals can be an important source of hope and thus contributes to the development of better care [59,60].

Understanding the prognosis of terminality means, in other words, accepting the approach of death. According to Prado et al. [48], no matter how good the reaction to this reality, it is unlikely that individuals have no feelings of anguish or frustration. When hope begins to dissipate and the consequences of the physical and clinical condition become evident, individuals begin to experience anguish and frustration, especially when they see their life projects or future possibilities being interrupted. This phase is when individuals experience greater fragility, not only physical, but also emotional and spiritual [61]. Several studies report distress and depression in almost half of terminally ill individuals and these conditions are significantly associated with the absence of support networks, caregiver overload and lack of assistance [62,63]. In contrast, sharing these moments of greatest need with care support networks is essential and suggests the possibility of a new perspective on their reality, allowing an understanding of death not as an end, but as a potential for other possibilities [64].

As in previous studies on this issue, our results highlight the positive effects of palliative care, through specialized support networks at home. In this sense, the support of palliative care teams has proved to be essential to alleviating feelings of anguish, depression and caregiver burden and other needs inherent to care [65,66].

Specialized care at home opens space for reflection on the weaknesses and concerns associated with EoL, and for reconfiguration of individual priorities, namely the need to enjoy time with family and do what brings comfort and well-being. Individuals come to understand finitude as an integral part of their condition, and therefore begin to reflect more naturally and clearly upon the proximity of death [67,68].

Many individuals see the proximity of death as a “last chance”, and therefore want to make it worthwhile, beginning to emphasize previously undervalued details and equating new priorities and meanings [39,48].

When the person becomes aware of their EoL, they have time to make arrangements regarding certain matters, whether material or spiritual, to remember important moments, and to express wishes and recommendations. In this plurality of meanings of life and death, there is a common denominator: the awareness of the end. The nature of palliative care patients’ transitions and reactions to transitions may vary, thus our results and adaptations may differ from those of other populations [69]. Our study revealed that the relational and interactive dimensions are significant in the transition experience of terminally ill persons and their families.

In addition, the transition from hospital to home care exposes numerous constraints, mainly related to the type of specialized responses in palliative care and the support networks available [40]. However, there is a parallel universe of variables that must be considered when preparing for death, with emphasis on care associated with anticipatory grief as part of the normal dying process [12]. Terminally ill people and their loved ones should prepare for death by grieving the various losses implicit in the death [70]. In this sense, there should be an investment in “grief-based interventions used by palliative care teams to help the patient/family comfortably journey through this final stage of life” [70] (p. 2).

### 4.1. Strengths and Limitations

Grounded theory was chosen in this study because the aim was to provide empirical knowledge in the absence of prior hypotheses. Using its paradigm structure, the model of participant experiences could be presented in an integrated and structured way. The findings were consistent with those of previous studies, bolstering the study’s credibility and dependability, and the transference of its findings to different groups in various contexts. However, although we attempted to offer an exhaustive account of events, there were certain limitations. We did not include caregivers of newly diagnosed patients or those in the grief stage. As a result, certain details may have been neglected. None of the participants expressed a desire to read their interview transcripts. The paucity of representation in transitions from and to care homes hampered the opportunity to study and compare the views of those experiencing care home transitions with other forms of transitions. Data collection was restricted due to time constraints and physical distancing imposed by the COVID-19 pandemic. For that reason, the study sample is small and relatively homogeneous, and the findings must be interpreted accordingly. Finally, despite many efforts, certain culturally specific meanings and connotations of words may have been lost in translation.

### 4.2. Implications for Practice

Transitions between care facilities faced by EoL patients with severe illnesses and their family caregivers highlight the need for person-centred care and continuity, implying that improved integration of palliative care across venues is essential [27]. Patients and their families are both at risk during transitions, which could lead to an increase in family stress. Transitions can be made more difficult by the difficulty of the discharge procedure and the fragmented character of the healthcare system. There is a need for organizational and national policy measures that promote and ease EoL transitions, while respecting the unique needs of patients receiving palliative care in the home and community environment [15].

Improvements in communication between teams and across organizations, clarification of accountability as patients move between settings, standardization of discharge procedures, and continuing training for health professionals on psychosocial and spiritual support, communication skills, and information sharing should be developed and integrated to ensure safe transitions for patients and their families [27]. Another potential strategy to improve transitions is to create the role of nursing care coordinator, dedicated to the coordination and management of transitions [15].

Collaborative integrated palliative care projects might increase the quality of palliative care in Brazil by optimizing continuity of care experiences. However, these programs must be further integrated with other health care professionals participating in the care networks of patients with advanced illnesses and their family carers. These findings might be used by practitioners, researchers and policymakers to improve integrated palliative care and the experiences of patients with life-threatening conditions and family caregivers [24].

The findings obtained through this CGT are an important starting point for other works, namely improving care of patients and family members who need palliative care at home. Moreover, the findings deepen our perspective of the biopsychosocial aspects and needs of patients and families, namely those which allow them to be authors of their own narrative until the moment of death. A compassionate, welcoming, reliable, and competent perspective by health professionals is necessary, one which encourages a qualitative appreciation of life, ensures security and autonomy, and fosters dignity in death.

## 5. Conclusions

The present study provides substantive theoretical insights into the terminally ill process during hospital-to-home transitions in the Brazilian cultural context. In the final stage of life, patients and their families express a preference for receiving palliative care, and consequently death, at home. Based on the findings, this choice is reflected in environments where there is a professional and social support structure, which generates security and comfort in times of greatest suffering. The return to the family and social context and feeling safe in their intimacy are factors the home environment can provide, and individuals who share this reality are able to better understand their finitude and accept it as an integral part of their condition. Throughout the EoL process, reflecting and reframing the primordial values of their lives and what makes sense to them allows them to clarify the world around them. Giving voice to individuals who experience the process of terminality at home guarantees the right to a dignified death, recognizing the importance of identity, continuity of care, and emotional and practical support. For health professionals, it is an opportunity to provide those who face this process with structured and humanized care in the defence of human dignity.

## Figures and Tables

**Table 1:** Sample characteristics (*n* = 21 participants).

Characteristics	Patients [PT] (*n* = 11)	Family Caregivers (*n* = 10)
Age (years) Mean (SD; range)	62 (19, 96; 31–84) years	50 (10, 85; 35–68) years
**Sex**		
Female	7	9
Male	4	1
**Primary clinical condition**		NA
Neurodegenerative Disease	3 PT with Dementia/Senility; and 2 PT with Central Nervous System Disease	NA
Cancer	2 Breast; 1 Prostate; 1 Intestine; 1 Ovary	NA
Traumatic Injury	1 PT with after-effects of an automobile accident	NA
**Length of the caregiving role**		
>3 months to ≤1 year	NA	1
>1 year to ≤2 years	NA	3
>2 years to ≤3 years	NA	6

NA = Not applicable.

## Data Availability

All data generated or analysed during this study are included in this article.
